# Getah Virus: A New Contaminant in Veterinary Vaccines

**DOI:** 10.3390/vetsci12020082

**Published:** 2025-01-23

**Authors:** Pin-Pin Chu, Sheng-Nan Chen, Xia Zhou, Zu-Zhang Wei, Shao-Lun Zhai

**Affiliations:** 1 Department of Swine Diseases, Institute of Animal Health, Guangdong Academy of Agricultural Sciences, Guangdong Provincial Key Laboratory of Livestock Disease Prevention, Scientific Observation and Experiment Station of Veterinary Drugs and Diagnostic Techniques of Guangdong Province, Ministry of Agriculture and Rural Affairs, Guangzhou 510640, China; cpp1900@163.com (P.-P.C.); xiazhou698868@163.com (X.Z.); 2 Laboratory of Animal infectious Diseases and Molecular Immunology, College of Animal Science and Technology, Guangxi University, Nanning 530005, China; chen.shengnan@sslab.com.cn (S.-N.C.); 3 Guangzhou Sino-Science Gene Testing Service Co., Ltd., Guangzhou 510700, China

**Keywords:** porcine reproductive and respiratory syndrome virus, Getah virus, live vaccine contaminant, vaccination infection, exogenous pathogens

## Abstract

Mycoplasma, reticuloendotheliosis virus (REV), avian leukosis virus (ALV), chicken infectious anemia virus (CIAV), bovine polyomavirus (BPV), bovine viral diarrhea virus (BVDV), and porcine circovirus (PCV) are considered common contaminants in live veterinary vaccines against Newcastle disease virus (NDV), fowlpox virus (FPV), infectious bursal disease virus (IBDV), classical swine fever virus (CSFV), pseudorabies virus (PRV), and porcine reproductive and respiratory syndrome virus (PRRSV). In the past five years, Getah virus (GETV), an arbovirus affecting many farming mammals, was reported as a new contaminant in live PRRSV vaccines in two previous studies, which arouses our considerable interest. Therefore, in this paper, we aim to analyze and discuss the source, biological hazard, and genomic characteristics of these contaminating GETV strains further.

## Simple Summary

Vaccines are essential for the prevention and control of infectious diseases in livestock farming. Among these, live veterinary vaccines play an important role. The production of live vaccines requires high-level biosafety, toxicity and potential contaminants should be closely monitored. Unfortunately, other viral contaminants in commercial live-attenuated vaccines against a multitude of viruses have been discovered, which are difficult to detect and can cause huge losses. Similar situations have occurred in commercial live-attenuated vaccines against porcine reproductive and respiratory syndrome virus (PRRSV), which arouses our considerable interest.

## 1. Introduction

Veterinary vaccines are important and economic measures for controlling animal infectious diseases. Safety and efficacy are important criteria for evaluating the quality of vaccines. The contamination of exogenous pathogens in live vaccines can result in the spread of exogenous pathogens and severe diseases in vaccinated animals [1,2]. Therefore, surveillance of exogenous pathogens is crucial during the development of vaccines.

Previously, mycoplasma, REV, ALV, CIAV, BVDV, FERV, and PCV have been considered common contaminants in live veterinary vaccines against NDV, FPV, IBDV, CPV, and CSFV [3,4,5,6,7,8,9,10,11,12,13,14,15,16,17,18,19,20].

PRRSV is a very important pathogen in the world swine industry and can infect pigs of all ages and cause serious respiratory and reproductive disorders, delayed growth, and even death. Moreover, PRRSV infection often causes immunosuppression, which provides opportunities for co-infection or secondary infection with other pathogens. PRRSV, with high variability in the genome, undergoes rapid evolution through mutation and recombination. PRRSV variants, including highly pathogenic PRRSV-like PRRSV, NADC30-like PRRSV, and NADC34-like PRRSV, are widely distributed around the world. All of these make prevention and control difficult [21,22,23]. There are currently two main kinds of vaccines against PRRSV available. One is an inactivated vaccine, and another is an attenuated live vaccine. Although the attenuated live vaccine has a better immune effect than the inactivated vaccine, the attenuated live vaccine can easily be contaminated by exogenous pathogens, including BVDV [17]. This might result in outbreaks of BVDV via vaccination with the live PRRSV vaccine in pig herds [17].

GETV, an arbovirus, can infect many kinds of animals and even humans, resulting in significant economic losses and death [24,25,26]. The original GETV strain MM2021 was isolated from mosquitoes in Malaysia in 1955, and GETV infection was first reported in diseased Japanese racehorses (16.48%, 70/455) in 1978. Seven years later, in 1985, a GETV outbreak occurred in a pig farm in Japan and led to death in newborn piglets. Since then, GETV infection has occurred in pigs and other animals such as cattle, foxes, and red pandas in different regions and countries, leading to substantial economic losses [27,28,29]. Equally, serological surveys have revealed the presence of GETV antibodies in humans. Expansion of the host range and geographical distribution of GETV has posed potential threats to public health. Although GETV can cause reproductive failure in sows and high mortality in neonatal piglets and result in huge economic losses in pig breeding, it is easily neglected because its symptoms are similar to those of infections caused by other viruses, such as PRRSV. In the past five years, there have been two studies to report the contamination of GETV in a live PRRSV vaccine [30,31]. GETV, as a new contaminant, should be given considerable attention. In this paper, we aim to analyze and discuss the source, hazard, and genomic characteristics of the contaminating GETV strains further.

## 2. Evidence of Contamination with GETV in Live PRRSV Vaccines

The first evidence of contamination by GETV in a live PRRSV vaccine was recorded by a Chinese research team in 2020 [30]. In fact, the collection time of the live PRRSV vaccines was the year of 2017. According to the description in the reference above, after immunization with the live PRRSV vaccine, abortions in pregnant sows in a pig farm increased. The author suspected that there may be other potential pathogenic pathogens in the vaccine. Firstly, Marc-145 cells were used to isolate potential contaminant pathogens, and the results showed that the cytopathic effect (CPE) significantly differed from that caused by PRRSV strains. More cell lines, including Vero, PK-15, BHK-21, and human hepatocellular carcinoma cells (HepG-2), were used, showing a similar phenomenon. And then, a specific polymerase chain reaction (PCR) using specific primers (the forward primer 5′-ACCGAAGAAGCCGAAGAA-3′ and reverse primer 5′-GCACTCRAGGTCATACTTG-3′) identified the presence of GETV nucleic acid in the vaccine samples. In addition, an immunofluorescence assay (IFA), transmission electron microscopy (TEM), and genome sequencing (of the isolate of GETV-V1) were used to confirm GETV as the contaminant in the live PRRSV vaccines. Further serological investigations using a self-designed enzyme-linked immunosorbent assay (ELISA) method based on the GETV Cap protein showed that a 100% antibody-positive rate occurred in the sow farms vaccinated with the live PRRSV vaccine, and it was higher than that (96.7%) in sow farms without vaccination with the live PRRSV vaccines. This result showed that GETV infection might have occurred on all of the sow farms at some points in the past. To further assess the side effect of GETV as a contaminant in the live PRRSV vaccine, animal experiments should be conducted in two groups of animals: one group should receive the live PRRSV vaccine, and the other should be served as a control [30].

In 2023, another Chinese research team recorded the second piece of evidence of contamination by GETV in live PRRSV vaccines [31]. One of several commercially modified live PRRSV vaccines from the same batch was found to be GETV-positive via specific RT-PCR. Subsequently, a new GETV isolate, named BJ0304, was isolated in ST cells. Animal experiment showed that the BJ0304 strain did not cause clinical signs and obvious histopathological changes in mice. Only testis and kidney samples from GETV-inoculated mice were positive by 7 and 14 days post-inoculation (dpi). This reveals the low pathogenicity of the BJ0304 strain [31].

## 3. Genomic Comparison Between Vaccine-Contaminated GETV Strains and Other GETV Strains

### 3.1. Genomic Comparison of GETV-V1 and Other GETV Strains

**Figure 1:**
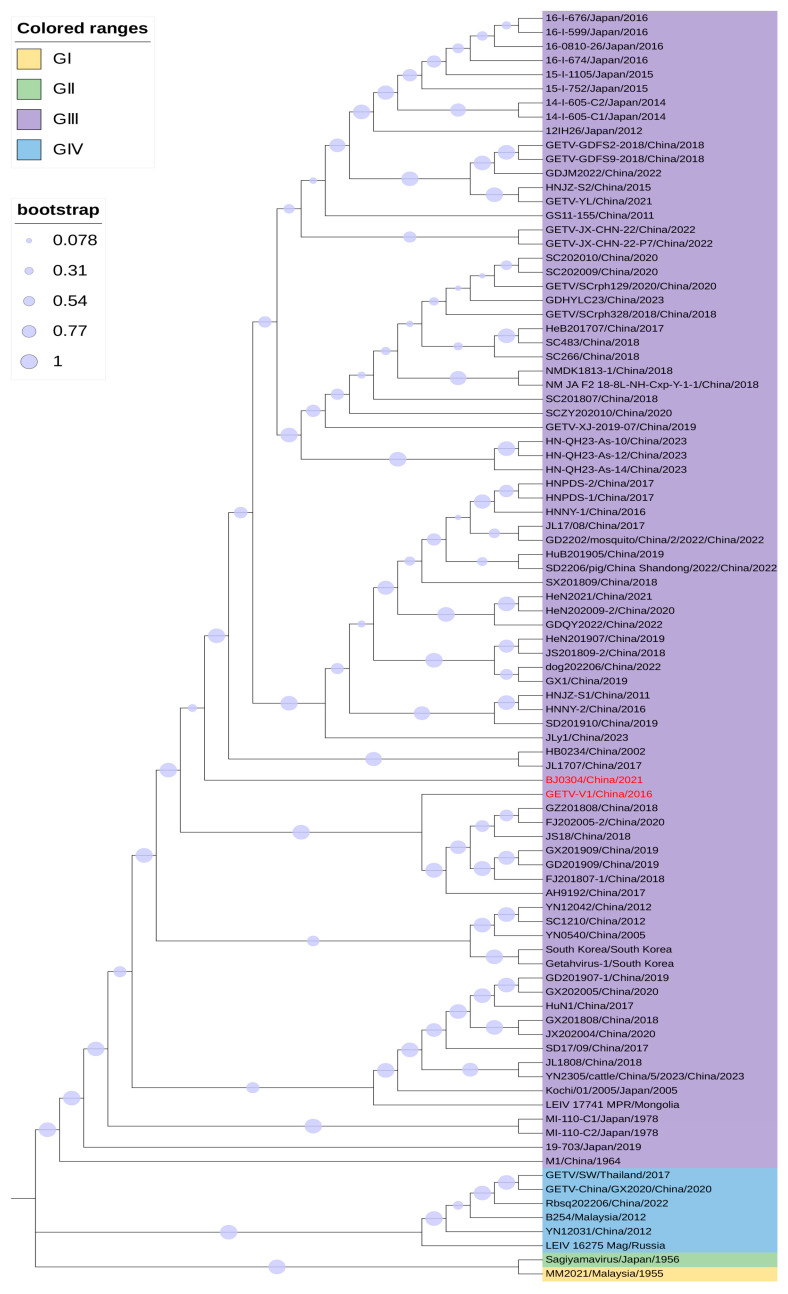
Phylogenetic analysis of GETVs. A phylogenetic tree based on complete genomic sequences was constructed using the maximum likelihood method with 1000 replicates and the GTR+G model in MEGA (version 11.0.13).

The genome sequences of two above-mentioned vaccine-contaminated GETV strains were collected from the GenBank database. Meanwhile, 88 complete or nearly complete genomic sequences from the GenBank database as of 21 May 2024 were also downloaded for sequence analysis. Among these, the GETV-V1 strain had the highest full-length genome nucleotide similarity with a porcine-origin strain AH9192 (GenBank no. MG865965) isolated in 2017 (Table 1). A phylogenetic analysis based on the complete genome indicated that GETV-V1 belonged to group III (Figure 1). Its 5′ untranslated region (UTR) is relatively conservative, having 98.7% nucleotide similarity with most reference 5′ UTR sequences (Table 1). In terms of the non-structural polyprotein gene, the GETV-V1 strain had the highest nucleotide (99.5%) and amino acid (99.6%) similarity with a porcine-origin strain JS18 (GenBank no. MT210319) isolated in 2018. In terms of the structural polyprotein gene, it had the highest nucleotide (99.4%) similarity with the AH9192 and JS18 strains, while it had the highest amino acid (99.7%) similarity with the JS18 strain. Moreover, its 3′ UTR had the highest nucleotide similarity (99.5%) with that from AH9192. In short, the GETV-V1 strain was highly related to these porcine-origin GETV strains.

### 3.2. Genomic Comparison of BJ0304 and Other GETV Strains

A whole genome sequence analysis showed that BJ0304 had the highest similarity (99.1%) to six porcine-origin strains (HNJZ-S1 from 2011, GenBank no. KY363862; HNNY-1 and HNNY-2 from 2016, GenBank nos. MG865966 and MG865967; HNPDS-1 and HNPDS-2 from 2017, GenBank nos. MG865968 and MG865969; SD2206 from 2022, GenBank no. PP623164) and three mosquito-origin strains (JL1707 from 2017, GenBank no. MH722255; HB0234 from 2002, GenBank no. EU015062; and GS11-155 from 2011, GenBank no. ON828424) (Table 1). A phylogenetic analysis based on the complete genome indicated that BJ0304 belonged to group III (Figure 1). Its 5′ UTR is quite conservative, having 100% nucleotide similarity with most reference 5′ UTR sequences. In terms of the non-structural polyprotein gene, BJ0304 had the highest nucleotide (99.3%) similarity with two porcine-origin strains (GenBank nos. AY702913 and NC_006558) isolated from South Korea, while at the amino acid level, it shared the highest similarity (99.5%) with three porcine-origin strains (HNNY-2 from 2016; HNPDS-1 and HNPDS-2 from 2017), one red-panda-origin strain (SCrph328 from 2018, GenBank no. MZ357111), and one mosquito-origin strain (NMDK1813-1 from 2018, GenBank no. MW512827). In the structural polyprotein gene, it had the highest nucleotide (99.3%) and amino acid (99.8%) similarity with two porcine-origin strains (GenBank nos. AY702913 and NC_006558) isolated from South Korea. In addition, its 3′ UTR had the highest nucleotide similarity (99.2%) with that in HNJZ-S2 (a porcine-origin strain, GenBank no. KY363863); and JL1707 and HB0234 (two mosquito-origin strains). In general, BJ0304 was also highly close to these porcine-origin GETV strains.

## 4. Conclusions

To summarize, this study provides a detailed analysis of the sequences of two PRRSV vaccine-derived GETVs, revealing their high relevance to porcine-origin GETV strains. The contaminant of GETV in the live PRRSV vaccine may have come from raw and auxiliary materials of animal origin, such as trypsin and fetal bovine serum, used during the vaccine production process. There are several live pig vaccines against Japanese encephalitis virus (JEV), CSFV, PRV, and PRRSV that are widely used in Chinese swine herds. GETV exists in the PRRSV vaccine; this may just be the tip of the iceberg. In recent years, GETV cases have increased in Chinese pig herds [27,32,33,34,35,36,37], and these vaccines contaminated with GETVs might play an important role in its transmission. In the future, GETV should be included in live veterinary vaccine quarantines, and the detection of GETV in the raw and auxiliary materials and final vaccine products should be strengthened during vaccine manufacturing. Moreover, surveillance and vaccine development for GETV are also important in pig herds.

## Figures and Tables

**Table 1:** Similarity of two vaccine-contaminated GETV strains with 88 reference strains.

Strains	GETV-V1							BJ0304						
	Complete Genome	5′ UTR	Non-Structural Polyprotein		Structural Polyprotein		3′ UTR	Complete Genome	5′ UTR	Non-Structural Polyprotein		Structural Polyprotein		3′ UTR
	nt	nt	nt	aa	nt	aa	nt	nt	nt	nt	aa	nt	aa	nt
MM2021	95.2	97.4	95.4	98.7	95.1	98.1	93.5	95.3	98.7	95.5	98.5	95.2	98	94.2
Sagiyamavirus	97.1	98.7	97.4	99	97	98.4	94.6	97.3	100	97.6	99	97	98.4	95.3
M1	98	98.7	98.2	99	97.9	98.8	97.2	98.2	100	98.3	99	98	98.8	98
16-I-676	98.8	98.7	98.9	99.4	98.8	99.6	98.2	98.9	100	98.9	99.2	98.9	99.6	99
15-I-752	98.8	98.7	99	99.5	98.8	99.6	98.2	98.9	100	98.9	99.3	98.9	99.6	99
14-I-605-C2	98.8	98.7	99	99.5	98.8	99.6	98	98.9	100	99	99.3	98.9	99.6	98.7
12IH26	98.9	98.7	99	99.6	98.9	99.6	98.2	99	100	99	99.4	98.9	99.6	99
GETV-GDFS2-2018	98.7	98.7	98.8	99.4	98.7	99.4	98.2	98.8	100	98.8	99.2	98.8	99.4	99
HNJZ-S2	98.8	98.7	98.9	99.6	98.9	99.6	98.5	98.9	100	98.9	99.4	99	99.6	99.2
HNPDS-2	98.9	98.7	99.1	99.5	98.8	99.7	98	99.1	100	99.2	99.5	98.9	99.7	98.7
HNNY-2	98.9	98.7	99.1	99.5	98.9	99.7	98.2	99.1	100	99.1	99.5	99	99.7	99
JL17/08	98.9	98.7	99.1	99.5	98.9	99.6	98	99	100	99.1	99.4	98.9	99.6	98.7
HNJZ-S1	98.9	98.7	99.1	99.4	98.9	99.7	98.2	99.1	100	99.1	99.2	99	99.7	99
SC266	98.5	98.7	98.7	99.1	98.5	99.4	97.5	98.7	100	98.7	99	98.6	99.4	98.2
SC483	98.5	98.7	98.7	99.2	98.4	99.4	97.5	98.7	100	98.8	99.1	98.5	99.4	98.2
SC201807	98.7	97.4	98.9	99.5	98.7	99.6	95.5	98.8	98.7	98.9	99.4	98.8	99.6	96.2
JL1707	98.9	98.7	99.1	99.5	98.8	99.3	98.5	99.1	100	99.1	99.3	98.9	99.3	99.2
HB0234	98.9	98.7	99.1	99.3	98.8	99.3	98.5	99.1	100	99.2	99.2	98.9	99.3	99.2
JS18	/	/	99.5	99.6	99.4	99.7	/	96.6	/	99	99.3	98.6	99.2	/
AH9192	99.4	98.7	99.3	99.3	99.4	99.6	99.5	98.7	100	98.9	99.1	98.6	99.2	98.7
GETV-V1	100	100	100	100	100	100	100	99	98.7	99.1	99.3	98.9	99.5	98.2
YN12042	98.4	98.7	98.8	99.3	98.9	99.6	88.3	98.5	100	98.8	99.2	99	99.7	88.6
SC1210	98.7	98.7	98.9	99.4	98.6	99.5	96.7	98.8	100	98.9	99.3	98.7	99.5	97.5
YN0540	98.8	98.7	99	99.4	98.9	99.6	96.7	99	100	99	99.3	99	99.6	97.5
South Korea	98.2	98.7	99.2	99.5	99.2	99.6	70.2	98.4	100	99.3	99.4	99.3	99.8	70.6
GX201808	97.3	97.4	97.3	99	97.3	99	98.5	97.5	98.7	97.4	99	97.5	99	98.2
HuN1	97.5	98.7	97.4	99.2	97.8	99.2	97.7	97.7	100	97.5	99.1	97.9	99.2	98
SD17/09	97.7	98.7	97.7	99.3	97.6	99.2	98.5	97.8	100	97.8	99.3	97.7	99.2	98.7
JL1808	97.7	98.7	97.8	99.3	97.7	99.3	98.2	97.9	100	97.9	99.3	97.7	99.3	98.5
LEIV 17741 MPR	98.5	98.7	98.5	99.3	98.7	99.7	98.2	98.7	100	98.6	99.2	98.8	99.7	99
MI-110-C1	98.6	98.7	98.7	99.5	98.6	99.6	98	98.7	100	98.7	99.3	98.7	99.6	98.7
B254	/	/	96.3	98.4	96.3	98.4	/	/	/	96.4	98.2	96.1	98	/
YN12031	96.2	97.4	96.3	98.6	96.4	98.8	95.5	96.1	98.7	96.3	98.5	96.3	98.4	96
GETV/SW/Thailand/2017	96	98.7	96.3	98.8	95.8	98.6	94.7	95.9	100	96.3	98.7	95.6	98.3	95.2
LEIV 16275 Mag	97.4	97.4	97.6	99.2	97.5	99.2	96	97.4	98.7	97.7	99.1	97.6	99.2	96.7
GETV-JX-CHN-22	98.6	97.4	98.8	99.5	98.7	99.5	98	98.8	98.7	98.8	99.3	98.7	99.5	98.7
GETV-YL	98.7	98.7	98.9	99.5	98.8	99.7	98.2	98.8	100	98.9	99.3	98.8	99.6	99
GETV-XJ-2019-07	98.3	98.7	98.5	99.3	98.4	99.5	97.5	98.5	100	98.5	99.2	98.4	99.5	98.2
GETV/SCrph328	/	/	98.9	99.6	98.5	99.6	/	/	/	98.9	99.5	98.7	99.6	/
GS11-155	98.9	98.7	99.1	99.5	98.8	99.7	98.2	99.1	100	99.2	99.4	98.9	99.7	98.5
dog202206	0	98.7	98.8	99.5	98.5	99.3	0	0	100	98.9	99.4	98.5	99.2	0
Rbsq202206	96	98.7	96.3	98.7	95.9	98.5	94.7	95.9	100	96.4	98.6	95.7	98.2	95.2
GDQY2022	98.8	98.7	99	99.6	98.9	99.7	98	98.9	100	99	99.4	98.9	99.7	98.7
GDJM2022	98.5	97.4	98.7	99.5	98.5	99.3	98.2	98.6	98.7	98.6	99.3	98.6	99.3	99
SCZY202010	98.6	98.7	98.6	99.1	98.8	99.6	97.5	98.7	100	98.7	98.9	98.8	99.6	98.2
HeN2021	98.7	98.7	98.9	99.5	98.7	99.5	98	98.9	100	98.9	99.3	98.8	99.5	98.7
GD201907-1	/	/	97.2	98.7	97.5	99.1	/	/	/	97.3	98.7	97.6	99.1	/
GX201909	/	/	99.4	99.6	99.4	99.7	0	/	/	98.9	99.3	98.6	99.2	/
HeB201707	/	/	98.6	99	98.4	99.4	97.5	/	/	98.6	98.9	98.5	99.4	98.2
HeN201907	/	/	98.8	99.5	98.7	99.6	0	/	/	98.9	99.4	98.7	99.4	/
BJ0304	99	98.7	99.1	99.3	98.9	99.5	98.2	100	100	100	100	100	100	100
19-703	/	/	98.6	99.4	98.5	99.3	0	/	/	98.6	99.2	98.6	99.3	/
NMDK1813-1	98.3	98.7	98.8	99.6	98.5	99.6	85.8	98.4	100	98.9	99.5	98.7	99.6	86.5
GZ201808	/	/	99.4	99.5	99.4	99.6	/	/	/	99	99.3	98.6	99.2	/
GDHYLC23	98.3	97.4	98.8	99.5	98.5	99.6	89.4	98.5	98.7	98.8	99.4	98.6	99.6	90
GD2202/mosquito/China/2/2022	98.9	98.7	99.1	99.6	98.8	99.6	98	99	100	99.1	99.3	99	99.7	98.7
GETV-GDFS9-2018	98.7	98.7	98.8	99.4	98.7	99.4	98.2	98.8	100	98.8	99.2	98.8	99.4	99
HNPDS-1	98.9	98.7	99.1	99.5	98.9	99.7	97.5	99.1	100	99.2	99.5	98.9	99.7	98.2
HNNY-1	98.9	98.7	74.3	74.5	98.9	99.7	98	99.1	100	74.3	74.5	99	99.7	98.7
15-I-1105	98.8	98.7	98.9	99.4	98.8	99.6	98	98.9	100	98.9	99.2	98.9	99.6	98.7
16-I-674	98.8	98.7	98.9	99.4	98.8	99.6	98.2	98.9	100	98.9	99.2	98.9	99.6	99
16-I-599	98.8	98.7	98.9	99.4	98.8	99.6	98	98.9	100	98.9	99.2	98.9	99.6	98.7
14-I-605-C1	98.8	98.7	99	99.5	98.8	99.6	98.2	98.9	100	99	99.3	98.9	99.6	99
MI-110-C2	98.6	98.7	98.7	99.5	98.6	99.7	98	98.7	100	98.7	99.4	98.7	99.7	98.7
Kochi/01/2005	97.8	98.7	97.9	99.3	97.8	99.3	98	97.9	100	97.9	99.3	97.9	99.3	98.2
SC202010	98.4	98.7	98.6	99.1	98.4	99	97.5	98.6	100	98.6	98.9	98.5	99.2	98.2
GD201909	/	98.7	99.3	99.5	99.4	99.7	/	/	100	98.8	99.3	98.6	99.2	/
GX202005	/	/	97.3	98.9	97.6	98.8	/	/	/	97.4	98.8	97.6	98.7	/
JS201809-2	/	/	98.8	99.4	98.7	99.6	/	/	/	98.8	99.3	98.7	99.4	/
JX202004	/	/	97.2	98.9	97.4	99.1	98.5	/	/	97.3	98.9	97.5	99.1	98.7
SX201809	/	/	99	99.5	98.7	99.7	/	/	/	99	99.3	98.8	99.7	/
FJ202005-2	/	/	99.4	99.5	99.1	99.1	/	/	/	98.9	99.3	98.2	98.6	/
HuB201905	/	/	99.1	99.6	98.9	99.7	/	/	/	99.1	99.4	99	99.7	/
SD201910	/	/	98.9	99.4	98.9	99.7	97.7	/	/	98.9	99.2	99	99.7	98.5
SC202009	/	/	98.8	99.4	98.5	99.5	97.5	/	/	98.8	99.3	98.6	99.6	98.2
FJ201807-1	/	/	99.5	99.6	99.4	99.7	/	/	/	99	99.3	98.6	99.2	/
HeN202009-2	98.8	98.7	98.9	99.5	98.9	99.7	98	98.9	100	98.9	99.3	98.9	99.7	98.7
GX1	/	/	98.9	99.5	98.7	99.6	/	/	/	98.9	99.4	98.7	99.4	/
GETV/SCrph129/2020	/	/	98.8	99.5	98.5	99.6	/	/	/	98.8	99.4	98.6	99.6	/
NM,JA_F2_18-8L-NH-Cxp-Y-1-1	98.6	98.7	98.8	99.5	98.4	99.6	97.5	98.8	100	98.9	99.4	98.5	99.6	98.2
Getahvirus-1	98.2	98.7	99.2	99.5	99.2	99.6	70.2	98.4	100	99.3	99.4	99.3	99.8	70.6
16-0810-26	/	/	99	99.4	98.8	99.6	/	/	/	98.9	99.2	98.9	99.6	/
GETV-China/GX2020	/	96.1	95.6	98.3	95.1	98	/	/	97.4	95.6	98.2	95	97.7	/
YN2305/cattle/China/5/2023	97.7	98.7	73.3	74.3	97.7	99.3	98.5	97.9	100	73.3	74.3	97.7	99.3	98.7
JLy1	98.8	98.7	98.9	99.4	98.7	99.3	97.2	98.9	100	99	99.3	98.9	99.5	98
SD2206	98.9	98.7	99.1	99.6	98.9	99.7	98	99.1	100	99.1	99.4	99	99.7	98.7
HN-QH23-As-10	/	/	98.6	99.4	98.3	99.4	/	/	/	98.6	99.3	98.3	99.4	/
HN-QH23-As-12	/	/	98.5	99.4	98.3	99.5	/	/	/	98.6	99.3	98.4	99.5	/
HN-QH23-As-14	/	/	98.6	99.3	98.3	99.4	/	/	/	98.6	99.3	98.3	99.4	/
GETV-JX-CHN-22-P7	98.6	97.4	98.7	99.4	98.8	99.7	98	98.8	98.7	98.7	99.3	98.8	99.7	98.7

“/” indicates there being no available data due to incomplete sequences.

## Data Availability

The original contributions presented in this study are included in the article. Further inquiries can be directed to the corresponding authors.
